# Five Rootstocks for “Emperor” Mandarin Under Subtropical Climate in Southern Brazil

**DOI:** 10.3389/fpls.2021.777871

**Published:** 2021-12-20

**Authors:** Maria Aparecida da Cruz, Carmen Silvia Vieira Janeiro Neves, Deived Uilian de Carvalho, Ronan Carlos Colombo, Jinhe Bai, Inês Fumiko Ubukata Yada, Rui Pereira Leite Junior, Zuleide Hissano Tazima

**Affiliations:** ^1^Horticultural Research Laboratory, ARS, United States Department of Agriculture (USDA), Fort Pierce, FL, United States; ^2^Centro de Ciências Agrárias, Universidade Estadual de Londrina, Londrina, Brazil; ^3^Área de Fitotecnia, Instituto de Desenvolvimento Rural do Paraná, Londrina, Brazil; ^4^Centro de Ciências Agrárias, Universidade Federal Tecnológica do Paraná, Francisco Beltrão, Brazil; ^5^Área de Biometria, Instituto de Desenvolvimento Rural do Paraná, Londrina, Brazil; ^6^Área de Proteção de Plantas, Instituto de Desenvolvimento Rural do Paraná, Londrina, Brazil

**Keywords:** *Citrus* spp., scion-rootstock combination, tree growth, fruit quality, yield performance, Huanglongbing

## Abstract

Rootstocks modulate several characteristics of citrus trees, including vegetative growth, fruit yield and quality, and resistance or tolerance to pests, diseases, soil drought, and salinity, among other factors. There is a shortage of scion and rootstock cultivars among the combinations planted in Brazil. “Ponkan” mandarin and “Murcott” tangor grafted on “Rangpur” lime comprise the majority of the commercial mandarin orchards in Brazil. This low genetic diversity of citrus orchards can favor pest and disease outbreaks. This study aimed to evaluate the agronomic performance, Huanglongbing (HLB) tolerance, and fruit quality of “Emperor” mandarin on five different rootstocks for nine cropping seasons under the subtropical soil-climate conditions of the North region of the state of Paraná, Brazil. The experimental design was a randomized block, with six replications, two trees per block, and five rootstocks, including “Rangpur” lime, “Cleopatra,” and “Sunki” mandarins, “Swingle” citrumelo, and “Fepagro C-13” citrange. The evaluations included tree growth, yield performance, fruit quality, and HLB disease incidence. “Emperor” mandarin trees grafted on “Rangpur” lime and “Swingle” citrumelo had early fruiting and high yield efficiency. “Rangpur” lime also induced the lowest tree growth, but low fruit quality. Trees on “Swingle” citrumelo and “Fepagro C-13” citrange showed low scion and rootstock affinity and produced fruits with high total soluble solids (TSS), with a lower number of seeds for those from trees on “Fepagro C-13” citrange. “Cleopatra” and “Sunki” mandarins induced higher juice content, while fruits from trees on “Cleopatra” also had higher TSS/titratable acidity (TA) ratio. “Emperor” mandarin trees were susceptible to HLB regardless of the rootstocks. Overall, “Cleopatra” and “Sunki” mandarins, “Swingle” citrumelo, and “Fepagro C-13” are more suitable rootstocks for “Emperor” mandarin under Brazilian subtropical conditions than “Rangpur” lime.

## Introduction

Mandarins are the second most important group of commercial citrus produced worldwide, next to oranges. In 2019, the total mandarin production globally was 35 million tons, with almost three-quarters produced in Asia [Food Agricultural Organization (FAO), 2019]. China is the largest mandarin producer, followed by Spain, Turkey, Morocco, Egypt, the United States, and Brazil [Food Agricultural Organization (FAO), 2019]. In 2020, over one million tons of mandarin fruits were produced in Brazil [Instituto Brasileiro de Geografia e Estatística (IBGE), 2020]. The Brazilian mandarin production is concentrated in the states of São Paulo, Minas Gerais, Paraná, and Rio Grande do Sul [Instituto Brasileiro de Geografia e Estatística (IBGE), 2020].

Despite the global importance of the Brazilian citrus industry, there is a shortage of citrus scion and rootstock cultivars. Among the cultivated mandarins and mandarin-like, “Ponkan” (*Citrus reticulata* Blanc.) and “Murcott” tangor [*C. reticulata* × *C. sinensis* (L.) Osb.], grafted mostly on “Rangpur” lime (*C. limonia* Osb.), are the most extensively used combinations in Brazil, representing 80% of the total mandarin acreage (Stuchi et al., 2008; Pacheco et al., 2017). Although preferred by the Brazilian consumers, the commercialization of “Ponkan” is restricted due to its short postharvest life (Carvalho S. A. et al., 2019). Under this scenario, the genetic diversification of citrus orchards, for both scion and rootstock cultivars, is important to prevent disease and pest outbreaks, and to extend the harvest season, as well as to improve the commercial performance of the citrus species under different edaphoclimatic conditions, producing fruits of high yield and quality (Emmanouilidoua and Kyriacoub, 2017; Carvalho L. M. et al., 2019; Alfaro et al., 2021).

Rootstocks determine several traits of the citrus trees, including vegetative growth, longevity, water and nutrient absorptions, yield performance, fruit quality, and tolerance or resistance to biotic and abiotic stresses (Castle, 1995, 2010; Castle et al., 2010; Pestana et al., 2011; Legua et al., 2014). The rootstocks included in this study were chosen according to their performance in previous studies in different citrus-growing areas using multiple scions. “Rangpur” lime has been the most used rootstock in Brazil for several decades, and with “Swingle” citrumelo [*C. paradisi* Macf. × *Poncirus trifoliata* (L.) Raf.], are currently, the most important rootstock in the Brazilian citrus industry (Carvalho S. A. et al., 2019; Miranda et al., 2020). These rootstocks are between the 21 major world rootstocks in current use, along with “Cleopatra” (*C. reshni* Hort. ex Tan.) and “Sunki” (*C. sunki* Hort. ex Tan.) mandarins (Bowman and Joubert, 2020), also chosen to be evaluated in the present study. Although not extensively used, “Fepagro C-13” citrange [*C. sinensis* (L.) Osb. × *P. trifoliata* (L.) Raf.] was included in our study due to its higher horticultural performance reported in previous studies (Stenzel et al., 2003; Pompeu Junior and Blumer, 2014; Carvalho et al., 2021).

“Rangpur” lime is also used in other important citrus-growing areas, as China and India (Bowman and Joubert, 2020). This rootstock induces early fruiting and adequate production to the citrus trees (Pompeu Junior, 2005). In addition, “Rangpur” lime is compatible with most commercial citrus scions and is tolerant to the citrus Tristeza virus (CTV) (Pompeu Junior, 2005). Further, “Rangpur” lime is drought tolerant (Pedroso et al., 2014; Miranda et al., 2020). This last trait has gained more attention due to climate changes and the need for plants to adapt to a wide range of environmental conditions (Alfaro et al., 2021; Aparicio-Durán et al., 2021). However, the susceptibility of “Rangpur” lime to some diseases has raised concerns and need to search for new alternative rootstocks for the Brazilian citrus industry (Pompeu Junior and Blumer, 2014; Fadel et al., 2018; Carvalho L. M. et al., 2019; Carvalho S. A. et al., 2019; Carvalho et al., 2021).

“Swingle” citrumelo has become an alternative for rootstock diversification in several countries globally, including the United States, Spain, and Mexico (Castle et al., 2010; Cruz et al., 2019; Bowman and Joubert, 2020). Similar to “Rangpur” lime, “Swingle” citrumelo induces early fruiting and is resistant to CTV (Castle and Stover, 2000; Castle, 2010). Further, it is also resistant to nematodes, *Phytophthora nicotianae*, and citrus blight (Castle and Stover, 2000; Castle, 2010). “Cleopatra” mandarin has also been used in several citrus-growing areas. This rootstock induces excellent yields, vigorous growth, and shows tolerance to citrus blight, CTV, xyloporosis, and some abiotic stresses such as salinity, cold, and calcareous soils (Castle, 1987; Pompeu Junior, 2005). Similar to “Cleopatra,” “Sunki” mandarin tolerates salinity, citrus blight, CTV, and xyloporosis and produces high-quality fruits and vigorous trees (Pompeu Junior, 2005). “Fepagro C-13” citrange is mostly used in Southern Brazil. This rootstock enhances the fruit yield and quality of the scion, besides being tolerant to some detrimental diseases and cold (Leite Junior, 1992; Stenzel et al., 2003; Pompeu Junior and Blumer, 2014; Carvalho et al., 2021).

As mentioned above, rootstocks are the key to facing challenges in the citrus industry. Currently, Huanglongbing (HLB) has been a major challenge to citrus production globally. The disease seriously affects citrus fruit quality and yield. Fruits from HLB-infected trees are usually reduced in size, sometimes asymmetric, greener, and have lower total soluble solids contents (TSS), higher titratable acidity (TA), and lower TSS/TA ratio (Dagulo et al., 2010; Dala-Paula et al., 2018, 2019). At present, there is no cure for HLB-infected trees (Bergamin Filho et al., 2016; Bassanezi et al., 2020). Recent studies revealed that some *Citrus* relatives seem to be more tolerant to HLB, by not showing typical HLB symptoms despite being infected (Albrecht and Bowman, 2012; Albrecht et al., 2016; Stover et al., 2016; Alves et al., 2021; Aparicio-Durán et al., 2021). However, no true resistance to the disease is known in the genus *Citrus* so far (Stover and McCollum, 2011; Albrecht and Bowman, 2012; Bergamin Filho et al., 2016).

The rootstock may perform differently when grafted with different scions. “Emperor” (*Citrus reticulata* Blanc.) is early to mid-season mandarin grown, mainly in Australia (Ladaniya, 2008). This mandarin is moderately resistant to citrus canker caused by the bacterium *Xanthomonas citri* subsp. *citri* (*Xcc*), a detrimental disease for the Brazilian citrus industry, with fruits of orange-colored, smooth skin, and seedy (Ladaniya, 2008; Leite Junior, 2015). Western Australia is the largest mandarin producer in Australia and has climatic conditions similar to Southern Brazil, with maximum and minimum mean temperatures of 23 and 13°C, respectively [Bureau of Meteorology Western Australia (BOM), 2016]. “Emperor” may be a potential alternative for citrus scion diversification in Southern Brazil, as well as to other citrus-growing areas around the world, with a similar humid subtropical climate, such as Florida in the United States, East and South-Central China, and the coastal areas of Mexico. Accordingly, this study aimed to evaluate the influence of five rootstocks on the vegetative growth, yield performance, fruit quality, and HLB tolerance of “Emperor” mandarin under the humid subtropical climate of Southern Brazil.

## Materials and Methods

### Experimental Location

**FIGURE 1:**
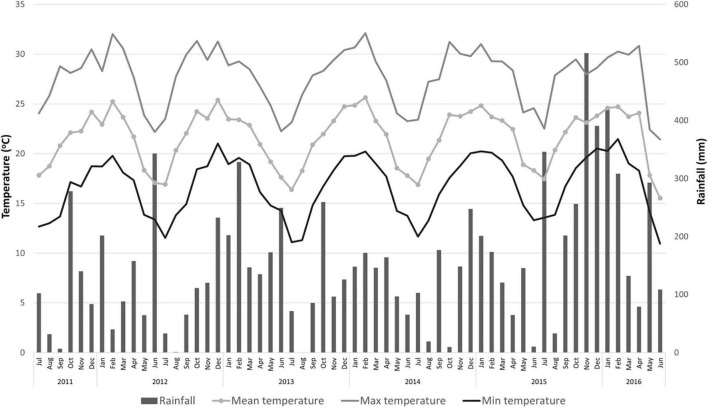
Rainfall, and mean, maximum and minimum temperatures for 2011 through 2015 period in Londrina, Paraná, Brazil. [Source: Instituto Agronômico do Paraná (IAPAR), 2018].

The experiment was conducted at the Experimental Station of the Instituto de Desenvolvimento Rural do Paraná (IDR-Paraná) in Londrina, Paraná, Brazil (Latitude 23° 21′ 34″ S; Longitude 51° 09′ 53″ W; and altitude of 585 m). The soil is classified as Oxisol Typic Hapludox, a clay soil with a pH of 5.0 or higher and a base saturation (by NH_4_OAc) of 35 % or less (U.S. Department of Agriculture, 1999), and the Köppen climate classification is Cfa (humid subtropical). The annual maximum and minimum mean temperatures are 27.3 and 16.1°C, respectively. The total annual rainfall is 1,639 mm (Figure 1) with a mean relative humidity of 70.5% [Instituto Agronômico do Paraná (IAPAR), 2018].

### Plant Materials and Management

The experimental orchard was planted in December 2005, at a tree spacing of 7.0 m × 4.0 m between and within rows, respectively, with a planting density of 357 trees ha^–1^. The orchard was not irrigated and weed control was performed periodically using an ecological rotary mower.

The experimental design was randomized blocks with five treatments (rootstocks), six blocks, and the data were collected from the two innermost trees of six trees per block. The rootstocks evaluated were “Rangpur” lime (*C. limonia* Osb.), “Cleopatra” mandarin (*C. reshni* Hort ex Tanaka), “Sunki” mandarin (*C. sunki* Hort ex Tanaka), “Swingle” citrumelo [*C. paradisi* Macf. cv. “Duncan” × *P. trifoliata* (L.) Raf.], and “Fepagro C-13” citrange [*C. sinensis* × *P. trifoliata* (L.) Raf]. Rootstock seeds and “Emperor” mandarin budwoods were obtained from the Citrus Active Germplasm Bank of the IDR-Paraná, in Londrina, Paraná, Brazil.

Trees were monitored periodically, and cultural practices were performed according to the recommendations for the state of Paraná, including preventative copper sprays to control citrus canker (*Xanthomonas citri* subsp. *citri*) and monthly insecticide applications to control the Asian citrus psyllid (*Diaphorina citri* Kuwayama) from 2014 up to 2016 [Instituto Agronômico do Paraná (IAPAR), 1992; Nunes et al., 2010]. The “Emperor” mandarin trees infected by the phloem-limited bacteria “*Candidatus* Liberibacter asiaticus,” pathogen of (HLB), were eliminated.

### Vegetative Growth

Vegetative growth was evaluated in the 2010 and 2016 seasons, after harvests. “Emperor” mandarin trees showed a broad-spread canopy with an oval shape, characteristic of the cultivar (Hodgson, 1967). The canopy volume was calculated based on tree height and canopy diameter, according to the equation proposed by Mendel (1956):


$$\text{CV}=\frac{2}{3}\times \pi \times {\text{CR}}^{2}\times \text{TH}$$

where **CV** = canopy volume (m^3^); ***CR*** = canopy radius (m); and ***TH*** = tree height (m).

The trunk circumference was determined at 10 cm above (TDA) and below (TDB) the graft union and converted to diameter. Based on these measurements, the ratio between the trunk diameter below and above the graft union (TDB/TDA) was calculated. No pruning was performed at any stage of the tree growth.

### Yield Performance

Fruit yield was determined annually in June, from 2008 to 2016 using a digital scale, and the results were expressed in fruit weight per tree. The cumulative yield was calculated by the sum of the annual yields. The yield efficiency of the trees was determined based on the ratio between fruit yield and canopy volume when the trees were 11 years old (2016). The alternate bearing index was determined according to Pearce and Dobersek-Urbanc (1967):


$$\text{ABI}=\left(\frac{1}{n-1}\right)\times \left[\left(\frac{a_{2}-a_{1}}{a_{2}+a_{1}}\right)+\left(\frac{a_{3}-a_{2}}{a_{3}+a_{2}}\right)+\ldots +\left(\frac{a_{\left(n\right)}-a_{\left(n-1\right)}}{a_{\left(n\right)}+a_{\left(n-1\right)}}\right)\right]$$

where **ABI** = alternate bearing index; ***n*** = number of years; and ***a_1_, a_2_, … a_(n)_, a_(n–1)_*** = yields of the corresponding years.

### Fruit Quality

The fruit quality was determined on 10 fruit samples per block. Samples were randomly collected at 1–2 m tree height in May for the seasons of 2012–2016, when the fruits reached maturity according to the international standards [Organization for Economic Co-operation and Development (OECD), 2010; Companhia de Entrepostos e Armazéns Gerais de São Paulo (CEAGESP), 2011]. The fruit height and diameter were measured with a Vernier digital caliper (Mitutoyo, ABS, Kawasaki, Kanagawa, Japan) and used to determine the fruit shape (FH FD^–1^). The fruits were weighted using a semi-analytic scale (total capacity of 15 kg) and classified according to the fresh citrus standards [Companhia de Entrepostos e Armazéns Gerais de São Paulo (CEAGESP), 2011]. The juice was extracted in a Croydon extractor (Croydon, Duque de Caxias, Brazil). The juice content (JC) was determined based on the following equation and the results were expressed as a percentage:


$$\text{JC}=\frac{\text{JW}}{\text{FW}}\times 100$$

where **JC** = juice content (%); ***JW*** = juice weight (g); and ***FW*** = fruit weight (g).

The seeds were manually extracted from each fruit and counted to determine the number of seeds per fruit. The TSS was determined with a digital refractometer (Atago Co., Ltd., Tokyo, Japan) using 0.3 ml of undiluted juice. Values were expressed in percentage (≈°Brix). The TA was determined by titrating 25 ml of juice with a standard solution of 0.1 N NaOH in an automatic titrator (TitroLine easy, Schott Instruments GmbH, Mainz, Rhineland-Palatinate, Germany). The acidity was expressed as the percentage of citric acid [Association of Official Analytical Chemists (AOAC) (2019)]. Then, the ratio between TSS and TA (TSS/TA) was used as the maturity indicator. The technological index (TI) or the amount of TSS per 40.8-kg box of fruits was determined according to the equation proposed by Di Giorgi et al. (1990).


$$\text{TI}=\frac{\text{TSS}\times \text{JC}\times 40.8}{10000}$$

where ***JC*** = juice content (%); and **40.8** = weight of the citrus industry standard box.

### Incidence of Huanglongbing

The experimental orchard was periodically monitored by a trained crew. Trees were visually screened for the presence of typical HLB symptoms, such as asymmetric mottling and thickening of veins in mature leaves. The first symptoms of HLB in the orchard were detected in 2014. The presence of HLB-associated bacterium, *“Candidatus* Liberibacter asiaticus” (*C*Las), was confirmed by PCR. In 2014 and 2015, a 12-leaf sample per tree was collected from the two innermost trees of each block, and DNA extraction was performed according to the protocol described by Murray and Thompson (1980). *C*Las was detected by the conventional PCR technique, using the primers A2 and J5, specific to *C*Las (Hocquellet et al., 1999). The PCR test was performed using the protocol described by Bagio et al. (2016). The DNA bands were visualized under ultraviolet light (L-PIX EX, Loccus do Brasil Ltda, Cotia, Brazil). Once the presence of the *C*Las was confirmed, the HLB-infected trees were marked and eliminated after the harvest season every year, as the eradication of HLB-symptomatic trees is mandatory in Brazil. The rate of HLB infection was expressed as the percentage (%) of diseased trees.

### Data Analyses

The experimental design was a randomized block, with five treatments (rootstocks) and six blocks. The data were tested for normal distribution and equal variance at *p* ≤ 0.05. Square root transformations were used for all data that did not follow the normal distribution. All data were evaluated by ANOVA followed by the comparison of the means according to Tukey’s test (*p* ≤ 0.05). Fruit quality parameters were assessed in a randomized block design with a factorial arrangement, main factor 1: five rootstocks × main factor 2: five cropping seasons, the interaction between these factors was evaluated for each parameter. The statistical analyses were conducted using the *R* version 4.1.0 (The R Foundation for Statistical Computing, Vienna, Austria) and the ExpDes package (Husson et al., 2017).

## Results

“Emperor” mandarin trees grafted on all evaluated rootstocks did not show any significant differences (*p* ≤ 0.05) in height, canopy diameter, and volume at the beginning of the trial (Table 1). However, in 2016, the trees grafted on “Rangpur” lime had smaller tree height and canopy volume than those on the other rootstocks, though did not differ from trees on “Fepagro C-13” for canopy volume (Table 1).

**TABLE 1:** Vegetative growth of “Emperor” mandarin trees grafted on five different rootstocks for the 2010 and 2016 cropping seasons. Londrina, Paraná, Brazil.

Rootstock	Tree height		Canopy diameter		Canopy volume		TDB^2^ (cm)		TDA^2^ (cm)		TDB/TDA^3^	
	(m)		(m)		(m^3^)							
						
	2010	2016	2010	2016	2010	2016	2010	2016	2010	2016	2010	2016
“Rangpur” lime	2.2 a^1^	2.7 b	2.7 a	3.9 a	8.3 a	21.9 b	32.2 b	47.6 c	24.6 ab	35.0 bc	1.3 b	1.4 c
“Cleopatra” mandarin	2.1 a	3.1 a	2.6 a	4.2 a	7.9 a	29.4 a	31.9 b	52.4 c	23.2 bc	37.1 ab	1.4 b	1.4 c
“Sunki” mandarin	2.4 a	3.1 a	2.8 a	4.2 a	9.7 a	29.0 a	33.4 b	55.0 bc	25.5 a	40.3 a	1.3 b	1.4 c
“Swingle” citrumelo	2.3 a	3.0 a	2.7 a	4.3 a	9.0 a	29.0 a	40.1 a	69.0 a	22.6 cd	33.6 cd	1.8 a	2.1 a
“Fepagro C-13” citrange	2.3 a	2.9 a	2.6 a	4.0 a	8.0 a	24.7 ab	35.5 ab	60.0 b	21.3 d	31.4 d	1.7 a	1.9 b
CV (%)^4^	6.50	4.70	7.44	5.28	16.56	11.68	7.86	7.22	4.29	5.12	7.97	4.85
Block	0.111^ns^	0.047*	0.494^ns^	0.165^ns^	0.224^ns^	0.060^ns^	0.270^ns^	0.062^ns^	0.266^ns^	0.027^ns^	0.554^ns^	0.008**
Rootstock	0.097^ns^	0.000***	0.582^ns^	0.064^ns^	0.263^ns^	0.002**	0.000***	0.000***	0.000***	0.000***	0.000***	0.000***

^1^Means followed by the same letter in the column did not differ according to Tukey’s test (p ≤ 0.05). ^2^Trunk diameters were based on trunk circumference measured 10 cm below (TDB) and above (TDA) the graft union. ^3^TDA/TDA, the ratio between scion and rootstock trunk diameter. ^4^Coefficient of variation (CV). p-value: ^ns^, non-significant, *p ≤ 0.05; **p ≤ 0.01; ***p ≤ 0.001.

The trees on “Swingle” citrumelo had the largest TDB in 2010 and 2016 (Table 1). Trees on “Fepagro C-13” citrange showed the smallest TDA in both evaluated years (Table 1). While trees on “Sunki” mandarin showed the largest TDA on both evaluations (Table 1). Furthermore, the TDB/TDA was significantly higher for the trees on “Swingle” and “Fepagro C-13,” compared with those on the other rootstocks in both seasons (Table 1).

The fruit yield of the “Emperor” mandarin trees grafted on the different rootstocks had a wide fluctuation across the experimental period (Table 2). In the first harvest, trees on “Rangpur” lime and “Swingle” citrumelo had higher yields than the other rootstocks, indicating earliness in fruit production (Table 2). The trees on “Swingle” citrumelo had the highest yields per tree in almost all seasons, except for 2010 (Table 2). The cumulative yield of the “Emperor” mandarin trees were not affected by the rootstock (Table 2). The yield efficiency was higher for trees grafted on “Rangpur” lime and “Swingle” citrumelo than those on other rootstocks (Table 2). “Fepagro C-13” citrange induced the lowest yield efficiency to “Emperor” mandarin (Table 2). The alternate bearing index of the “Emperor” mandarin trees was not affected by the rootstock, and the values ranged from 0.32 up to 0.50 (Table 2).

**TABLE 2:** Annual and cumulative yields, relative yield, yield efficiency, and yield alternate bearing index of “Emperor” mandarin trees grafted on five different rootstocks through nine consecutive cropping seasons (2008–2016) in Londrina, Paraná, Brazil.

Season	Yield (kg tree^–1^)					CV^2^ (%)	*p*-value	
	“Rangpur” lime	“Cleopatra” mandarin	“Sunki” mandarin	“Swingle” citrumelo	“Fepagro C-13” citrange		Block	Rootstock
2008	39.7 a^1^	23.8 b	18.8 c	31.1 a	17.2 c	22.99	0.012*	0.000***
2009	38.8 a	48.1 a	60.6 a	61.8 a	44.8 a	36.03	0.082^ns^	0.166^ns^
2010	63.3 a	60.2 a	25.3 b	30.3 b	49.1 ab	36.49	0.697^ns^	0.002**
2011	46.6 b	86.1 a	93.6 a	79.2 a	75.9 a	18.61	0.492^ns^	0.000***
2012	131.5 a	91.3 ab	80.0 b	118.7 ab	127.9 a	21.56	0.641^ns^	0.003**
2013	21.5 b	65.1 a	83.0 a	47.9 ab	69.3 a	35.54	0.378^ns^	0.000***
2014	24.4 c	34.9 bc	35.5 bc	51.4 ab	54.9 a	25.05	0.031*	0.000***
2015	29.6 b	79.3 a	69.0 ab	47.9 ab	27.0 b	56.67	0.729^ns^	0.016*
2016	78.4 ab	64.8 b	69.8 b	100.4 a	33.1 c	23.95	0.521^ns^	0.000***
Cumulative yield	473.8 a	553.6 a	535.5 a	568.7 a	499.3 a	14.00	0.978^ns^	0.192^ns^
Yield efficiency (kg m^–3^)^3^	3.7 a	2.2 b	2.4 b	3.5 a	1.4 c	26.16	0.375^ns^	0.000***
Alternate bearing index	0.39 a	0.32 a	0.50 a	0.40 a	0.40 a	14.61	0.798^ns^	0.196^ns^

^1^Means followed by the same letter in the row did not differ statistically according to Tukey’s test (p ≤ 0.05). ^2^Coefficient of variation (CV). ^3^The calculated yield efficiencies correspond only to the 2016 season. p-value: ^ns^, non-significant, *p ≤ 0.05; **p ≤ 0.01; ***p ≤ 0.001.

Significant interactions (*p* ≤ 0.001) were observed between harvest season and rootstock for all fruit quality parameters, except TSS (Tables 3, 4). Fruits from the trees on “Rangpur” lime showed an alternate in height, diameter, and weight across the evaluated period (Table 3). Fruits were smaller and lighter in the 2012, 2014, and 2016 seasons than those in the 2013 and 2015 seasons (Table 3). No differences in fruit height, diameter, shape, and weight were observed between the treatments for the 2016 season. In general, the “Emperor” fruits were smaller, lighter, and nearly round in shape for all scion-rootstock combinations in 2016, compared with fruits from the other seasons (Table 3).

**TABLE 3:** Physical quality of “Emperor” mandarin fruits from trees grafted on five different rootstocks, in Londrina, Paraná, Brazil.

Rootstock	Fruit height (FH, mm)					Fruit diameter (FD, mm)				
	2012	2013	2014	2015	2016	2012	2013	2014	2015	2016
“Rangpur” lime	45.0 cD^1^	59.1 aB	45.1 cD	65.2 aA	52.7 aC	58.7 dC	63.5 abB	55.6 cC	69.0 aA	58.5 aC
“Cleopatra” mandarin	53.3 bAB	53.0 bcAB	50.2 bcB	55.2 cA	52.6 aAB	66.3 abA	59.9 bBC	58.2 bcC	64.2 abAB	58.7 aC
“Sunki” mandarin	61.1 aA	52.0 cB	63.2 aA	53.4 cB	50.9 aB	70.7 aA	60.3 bB	70.4 aA	61.0 bB	58.2 aB
“Swingle” citrumelo	53.2 bB	58.6 abA	49.9 bcB	58.6 bcA	52.1 aB	65.2 bcA	63.5 aA	60.9 bAB	64.9 abA	58.4 aB
“Fepagro C-13” citrange	49.8 bcC	57.4 acA	56.6 bAB	61.5 abA	51.6 aBC	60.9 cdBC	63.0 abAB	66.5 aA	65.7 abA	57.8 aC
CV (%)	6.14					2.40				
Block	0.097^ns^					0.202^ns^				
Rootstocks	0.000***					0.000***				
Year	0.000***					0.000***				
Rootstock × Year	0.000***					0.000***				

**Rootstock**	**Fruit shape (FH/FD)**					**Fruit weight (g)**				
	**2012**	**2013**	**2014**	**2015**	**2016**	**2012**	**2013**	**2014**	**2015**	**2016**

“Rangpur” lime	0.77 cC	0.93 aA	0.81 cB	0.95 aA	0.90 aA	88.8 dC	120.5 abB	72.3 bC	161.8 aA	95.6 aC
“Cleopatra” mandarin	0.80 bcC	0.88 bcA	0.83 bcBC	0.86 bAB	0.89 aA	134.8 abA	104.4 bBC	150.1 aA	127.9 bcAB	97.0 aC
“Sunki” mandarin	0.86 aB	0.86 cB	0.91 aA	0.87 bAB	0.89 aAB	152.5 aA	102.2 bB	156.2 aA	112.5 cB	96.6 aB
“Swingle” citrumelo	0.82 bB	0.89 acA	0.82 bcB	0.90 abA	0.89 aA	121.3 bcA	130.9 aA	95.0 bB	130.7 bcA	95.6 aB
“Fepagro C-13” citrange	0.82 bC	0.91 abA	0.86 bB	0.94 aA	0.89 aAB	100.5 cdBC	119.0 abAB	131.9 aA	137.7 abA	93.3 aC
CV (%)	1.59					13.13				
Block	0.046*					0.069^ns^				
Rootstocks	0.000***					0.000***				
Year	0.000***					0.000***				
Rootstock × Year	0.000***					0.000***				

**Rootstock**	**Number of seeds**					**Juice content (%)**				
	**2012**	**2013**	**2014**	**2015**	**2016**	**2012**	**2013**	**2014**	**2015**	**2016**

“Rangpur” lime	26.3 aA	18.8 aB	25.1 aA	18.7 abB	20.2 bB	36.5 bB	42.7 aA	31.3 abC	37.4 bB	31.9 bC
“Cleopatra” mandarin	27.6 aA	19.7aB	21.7 abB	22.4 aB	21.9 abB	36.5 bBC	42.0 abA	34.4 aC	39.2 abAB	33.8 abC
“Sunki” mandarin	25.5 aA	20.1 aB	19.8 bB	22.0 aAB	19.6 bB	33.4 bB	41.1 abA	32.3 abB	41.8 aA	35.5 aB
“Swingle” citrumelo	23.5 aA	18.1 aB	21.9 abAB	21.1 aAB	22.0 abAB	35.3 bB	38.8 bcA	30.5 bC	40.7 abA	34.0 abB
“Fepagro C-13” citrange	24.0 aA	12.3 bC	19.6 bB	15.8 bBC	25.0 aA	40.7 aA	36.7 cB	31.9 abC	40.6 abA	34.5 abBC
CV (%)^2^	12.88					5.89				
Block	0.768^ns^					0.185^ns^				
Rootstocks	0.000***					0.060^ns^				
Year	0.000***					0.000***				
Rootstock × Year	0.000***					0.000***				

^1^Means followed by the same lowercase letters in the column or uppercase letters in the row did not differ statistically according to Tukey’s test. ^2^Coefficient of variation (CV). p-value: ^ns^, non-significant, *p ≤ 0.05; ***p ≤ 0.001.

**TABLE 4:** Chemical quality of “Emperor” mandarin fruits of trees grafted on five different rootstocks, in Londrina, Paraná, Brazil.

Rootstock	Total soluble solids (TSS,%)					Titratable acidity (TA, %)				
	mean					2012	2013	2014	2015	2016
“Rangpur” lime	9.8 b^1^					0.91 bcB	0.92 bB	1.00 aB	1.12 bA	1.12 cA
“Cleopatra” mandarin	10.1 ab					0.78 dD	0.99 abC	0.87 cCD	1.30 aA	1.18 cB
“Sunki” mandarin	10.1 ab					0.85 cdC	1.08 aB	0.90 bC	1.38 aA	1.39 aA
“Swingle” citrumelo	10.4 a					0.99 abB	1.00 abB	1.06 aB	1.36 aA	1.30 abA
“Fepagro C-13” citrange	10.3 a					1.06 aBC	0.94 bC	0.97 abC	1.18 bAB	1.19 bcA
2012	11.2 A					–	–	–	–	–
2013	9.8 C					–	–	–	–	–
2014	10.6 B					–	–	–	–	–
2015	9.5 C					–	–	–	–	–
2016	9.7 C					–	–	–	–	–
CV (%)	5.13					7.06				
Block	0.656^ns^					0.349^ns^				
Rootstocks	0.000***					0.000***				
Year	0.000***					0.000***				
Rootstock × Year	0.141^ns^					0.000***				

**Rootstock**	**Ratio (TSS/TA)**					**Technological index TI (kg TSS box^–1^)**				
	**2012**	**2013**	**2014**	**2015**	**2016**	**2012**	**2013**	**2014**	**2015**	**2016**

“Rangpur” lime	12.0 bA	10.2 abB	9.9 bB	8.0 aC	8.6 aC	1.64 bA	1.64 abA	1.33 aB	1.56 aA	1.19 bB
“Cleopatra” mandarin	13.9 aA	10.1 abC	11.9 aB	7.5 abD	8.3 aD	1.62 bAB	1.70 aA	1.38 aC	1.51 aBC	1.37 abC
“Sunki” mandarin	13.3 aA	9.2 bC	11.8 aB	6.8 bD	6.8 bD	1.53 bAB	1.67 abA	1.39 aB	1.63 aA	1.35 abB
“Swingle” citrumelo	11.5 bA	9.8 abB	10.3 bB	7.2 abC	7.8 abC	1.63 bA	1.56 abAB	1.36 aB	1.61 aA	1.44 aB
“Fepagro C-13” citrange	11.0 bA	10.6 aA	11.6 aA	8.1 aB	7.9 abB	1.90 aA	1.48 bBC	1.45 aBC	1.56 aB	1.31 abC
CV (%)^2^	7.3					8.18				
Block	0.369^ns^					0.146^ns^				
Rootstocks	0.000***					0.279^ns^				
Year	0.000***					0.000***				
Rootstock × Year	0.000***					0.000***				

^1^Means followed by the same lowercase letters in the column or uppercase letters in the row did not differ statistically according to Tukey’s test. ^2^Coefficient of variation (CV). p-value: ^ns^, non-significant, ***p ≤ 0.001.

“Emperor” fruits from all scion-rootstock combinations had height:diameter ratios above 0.86 in most of the evaluated seasons, indicating a nearly round shape (Table 3). In 2012 and 2014, fruits from trees grafted on all rootstocks, except on “Sunki” mandarin in both years and on “Fepagro C-13” citrange in 2012, were below 0.83, indicating a moderately oblate shape (Table 3). Fruits from the trees on “Rangpur” lime scored the lowest fruit weights in almost all cropping seasons (Table 3).

Fruits from trees on “Fepagro C-13” citrange showed a lower number of seeds in almost all evaluated seasons (Table 3). “Sunki” and “Cleopatra” mandarins induced the production of fruits with similar juice content across the evaluated period (Table 3). These fruits were among those with higher juice content in most of the evaluated years, except in 2012 (Table 3). On the other hand, fruits from the trees on “Rangpur” lime had low juice content in most of the seasons (Table 3). Fruits produced in the 2014 and 2016 seasons had lower juice content than those from the other seasons (Table 3).

As main effects, harvest season and rootstock were highly significant (*p* ≤ 0.001) for TSS over the five seasons, but no significant interaction between these factors was observed (Table 4). The TSS content was significantly higher in fruits produced by trees on “Fepagro C-13” and “Swingle” than those from trees on “Rangpur” (Table 4). Regarding the TSS per season, the values were relatively lower in 2013, 2015, and 2016 (Table 4). The TA was low for fruits from trees on all rootstocks in the first three seasons and increased in 2015 and 2016 (Table 4). “Swingle” citrumelo induced higher TA to “Emperor” mandarin fruits than the other rootstocks evaluated, being among those with the highest TA over the evaluated period (Table 4). On the other hand, “Rangpur” lime induced low TA to “Emperor” fruits in almost all seasons, except for 2014 (Table 4). The TSS/TA ratio was lower for fruits from the trees on all rootstocks in 2015 and 2016 compared to the TSS/TA ratio of fruits from the other seasons. “Emperor” mandarin fruits from trees on “Cleopatra” were among those with the highest TSS/TA ratio over the evaluated period (Table 4). Overall, the TSS and TSS/TA ratio were lower, and the TA was higher for fruits from trees on all scion-rootstock combinations in 2015 and 2016 compared with the other seasons (Table 4).

The TI varied through the seasons and was not influenced by the rootstocks in 2014 and 2015 (Table 4). However, there was a positive interaction between rootstock and cropping season (*p* ≤ 0.001). Fruits from all scion-rootstock combinations had lower TI in 2014 and 2016 than the fruits from the other seasons (Table 4).

The tree infection rate for HLB was 10% in 2014 and 30% in 2015, comprising a total of 40% of diseased trees (Table 5). The entire grove was eliminated in 2016, due to the high incidence of the disease. There was no statistical difference in the incidence of the disease between the evaluated rootstocks (Table 5).

**TABLE 5:** Incidence of Huanglongbing (HLB) disease on “Emperor” mandarin trees grafted on five different rootstocks in Londrina, Paraná, Brazil.

Rootstock	HLB-affected trees (%)		
	2014	2015	Total
“Rangpur” lime	0^1^	33	33
“Cleopatra” mandarin	17	33	50
“Sunki” mandarin	8	17	25
“Swingle” citrumelo	25	17	42
“Fepagro C-13” citrange	0	50	50
CV (%)^2^	21.19	23.97	23.47
^3^Block	0.78^ns^	0.91^ns^	0.97^ns^
^3^Rootstock	0.18^ns^	0.34^ns^	0.71^ns^

*^1^Means followed by the same letter in the column did not differ according to Tukey’s test (p ≤ 0.05). ^2^Coefficient of variation (CV). ^3^p-value: ^ns^, non-significant.*

## Discussion

The vegetative growth of the scion is directly affected by the rootstock, related to the genotype and its relationships (Auler et al., 2008). The vegetative growth of the “Emperor” mandarin trees observed in our study was similar to those of “Okitsu” satsumas and “Ponkan” mandarins, which also showed smaller tree size, i.e., height and canopy volume, when grafted on “Rangpur” lime and “Fepagro C-13” citrange, compared with those on the other rootstocks (Stenzel et al., 2003; Tazima et al., 2013). Similarly, the smallest growth pattern of the citrus trees grafted on “Rangpur” lime was reported for “Sunburst” and “Oneco” mandarins (Mourão Filho et al., 2007; Gonzatto et al., 2011), supporting the low vigor conferred by this rootstock to different scions.

The use of rootstocks that induce lower tree height and canopy volume allows the increase in plant density by area, which is a tendency in modern citrus production (Auler et al., 2008; Stover et al., 2008; Pompeu Junior and Blumer, 2009). High-density orchards maximize fruit quality and yield, decrease harvest costs, and facilitate crop management (Stover et al., 2008; Pompeu Junior and Blumer, 2009; Stuchi et al., 2012). In addition, higher densities orchards may improve profitability for farmers in HLB-endemic areas, under the removal of HLB-symptomatic trees (Moreira et al., 2019). In our study, no pruning was performed at any stage of the tree growth. However, in commercial orchards, scion-rootstock combinations with small vegetative growth may require less frequent pruning. This can result in less frequent emission of new shoots, which may contribute to a decrease in the attack of the Asian citrus psyllid (Stuchi et al., 2012).

The largest trunk diameter below the graft union reported for trees on “Swingle” citrumelo is a well-known characteristic conferred by this rootstock to several citrus species. Trees on “Swingle” citrumelo grow vigorously and show a trunk overgrowth near to the grafting union (Castle et al., 1988). Similar overgrowth has been observed for “Okitsu” satsuma mandarin and “Navelina,” “Jaffa,” “Cadenera,” and “Salustiana” sweet orange trees grafted on “Swingle” citrumelo (Tazima et al., 2013; Bacar et al., 2017; Domingues et al., 2018; Cruz et al., 2019; Carvalho et al., 2021).

The ratio between the trunk diameter below and above the graft union (TDB/TDA) may be an indication of scion and rootstock compatibility (Tazima et al., 2013), where indices close to one have been usually considered as the good affinity between them (Fadel et al., 2018). The highest TDB/TDA ratios of the “Emperor” trees were observed for those on “Swingle” citrumelo and “Fepagro C-13” citrange (Table 1). Similar TDB/TDA ratios were reported for other mandarins and sweet oranges grafted on the same rootstocks, such as “Marisol” clementine (Bassal, 2009), “Navelina” sweet orange (Cruz et al., 2019), and “Okitsu” satsuma mandarin (Tazima et al., 2013, 2014). However, the differences in trunk diameters between scion and rootstock may not be related to graft-incompatibility in some cases and may not always influence the horticultural performance of the scion (Fadel et al., 2018). Although the larger differences noticed in our study between scion and rootstock trunk diameters were for the trees on “Swingle” citrumelo and “Fepagro C-13” citrange, these trees did not show any symptoms of incompatibility or decay in the first eleven years after planting.

“Emperor” mandarin had early fruit production when the trees were grafted on “Rangpur” lime and “Swingle” citrumelo (Table 2). Trees grafted on these two rootstocks usually bear fruits at an early stage (Castle and Stover, 2000; Bowman and Joubert, 2020). This finding is in agreement with those reported for other citrus cultivars as “Okitsu” satsumas and “Oneco” mandarins, and the “Jaffa,” “Navelina,” and “Salustiana” sweet oranges (Gonzatto et al., 2011; Tazima et al., 2013; Bacar et al., 2017; Cruz et al., 2019; Carvalho et al., 2021). Rootstocks that induce early fruiting to the citrus trees are preferable, especially under the current HLB situation. HLB infection results in a short productive life of the citrus trees, reducing the economic life of the groves to less than 10 years due to the severity of the symptoms and the fast disease spread (Stover et al., 2008; Bové, 2012; Albrigo et al., 2019). Therefore, it is necessary that production reaches high levels early and maintains it for as long as possible during the orchard life (Stover et al., 2008).

The highest yields induced by “Swingle” citrumelo to the “Emperor” mandarin trees over the cropping seasons have also been reported previously for “Okitsu” satsuma mandarins, and “Navelina” and “Valencia” sweet oranges (Pompeu Junior and Blumer, 2011; Tazima et al., 2013; Cruz et al., 2019). However, the rootstocks did not influence the “Emperor” mandarin cumulative yield, though trees on “Swingle,” “Cleopatra,” and “Sunki” had yields 20, 17, and 13% higher than the trees on “Rangpur” lime, respectively (Table 2). The yield efficiency was higher for trees grafted on “Rangpur” lime and “Swingle” citrumelo than those on other rootstocks (Table 2). The yield efficiency is based on fruit production and canopy volume, “Rangpur” lime induced lower canopy volume to “Emperor” mandarin, which contributed to its high yield efficiency (Table 1). The use of rootstocks, which induce lower tree vegetative growth and high yield efficiency, enables the increase in plant densities per area, increasing fruit yield and facilitating harvest and crop management (Stover et al., 2008; Pompeu Junior and Blumer, 2009; Cruz et al., 2019).

We found no effect of the rootstocks on the alternate bearing of the “Emperor” mandarin trees (Table 2). Alternate bearing is common in mandarins and is characterized by irregular fruit production over the years (Siqueira and Salomão, 2016). The alternate bearing index ranges from 0 up to 1, where values closer to 0 indicate lower yield alternation (Tazima et al., 2014). Therefore, “Emperor” mandarin trees show low alternate bearing indices under subtropical conditions, ranging from 0.32 up to 0.50, regardless of the rootstock they were grafted on (Table 2). Similar results, with no effect of the rootstock, were reported for “Okitsu” satsuma, “Flagallo,” “Sunburst,” and “Span Americana” mandarins (Mourão Filho et al., 2007; Silva et al., 2013; Tazima et al., 2014). A high alternate bearing index usually results in small fruits with low quality in years of overproduction (Siqueira and Salomão, 2016). Therefore, the low alternate bearing revealed in our study for “Emperor” mandarin on multiple rootstocks can favor the production of fruits with better size and quality over the years.

Mandarins are produced primarily for the fresh fruit market (Albrigo et al., 2019). Although fresh citrus fruits must meet internal quality standards, the external appearance and fruit size are very important for consumer acceptance (Albrigo et al., 2019; Tarancón et al., 2021). Consumers’ preferences for fresh citrus fruits include seedless fruits with optimal size and shape and easily removable peel (Spreen et al., 2020). Generally, medium to large fruits provides higher returns to the growers (Hussain et al., 2013). The minimal mandarin fruit diameter accepted by the international fresh citrus market is 45 mm [Organization for Economic Co-operation and Development (OECD), 2010]. “Emperor” mandarin fruits of the trees on all evaluated rootstocks had larger diameters than the minimum standard (Table 3).

Fruit size is influenced by several factors, such as cultivar, rootstock, crop load, climate, and cultural practices (Albrigo et al., 2019). The variation on fruit size and weight observed in this study for fruits produced by trees grafted on “Rangpur” lime, may be related to the annual fruit load (Tables 2, 3). In 2012 and 2016, “Emperor” mandarin trees on “Rangpur” lime reached higher fruit yields, with fruits being smaller and lighter than those in the other seasons (Tables 2, 3). Crop load has a significant impact on citrus fruit size, with the final fruit size being inversely related to the number of fruits that reach maturity (Goldschmidt and Monselise, 1977; Guardiola and Lazaro, 1987; Agustí et al., 1999). This phenomenon is attributed to the competition between the developing organs for photosynthates and mineral elements (Albrigo et al., 2019). The high number of developing organs leads to strong competition for photosynthates and mineral elements and, consequently, to smaller final fruit sizes.

The fruit shape of mandarins may range from oblate to round (Goldenberg et al., 2018). A height:diameter ratio closer to 1 indicates a round shape, while a ratio around 0.65 indicates an oblate shape (Goldenberg et al., 2014, 2018). In this study, the “Emperor” mandarin fruits had shapes ranging from moderated oblate (0.77–0.83) to nearly round (0.86–0.95), depending on the rootstock and crop season (Table 3). Only fruits from the trees on “Sunki” mandarin presented a nearly round shape in all evaluations (Table 3).

The “Emperor” mandarin fruit weight was similar or heavier than those reported for this cultivar in a previous study, 90–100 g (Ladaniya, 2008). Except for those fruits from trees on “Rangpur” lime in the 2012 and 2014 seasons, that weighted less than 90 g (Table 3). Low fruit weight on fruits from trees grafted on “Rangpur” lime was also reported for the “Folha Murcha” sweet orange (Stenzel et al., 2005). In 2016, fruits from trees on all rootstocks had weights lower than 100 g (Table 3). Higher water supply during fruit development leads to an increase in fruit size and weight (Romero et al., 2006; Albrigo et al., 2019). However, the fruits of the 2016 season were smaller and lighter than those of the other seasons (Table 3), even with a high rainfall volume during fruit development (Figure 1). This was probably due to an increase in HLB infection in the grove at that season (Table 5). It is well known that HLB infection decreases the size and weight of citrus fruits (Dala-Paula et al., 2018, 2019).

“Emperor” mandarin fruits are seedy (Ladaniya, 2008). In this study, the number of seeds per fruit ranged from 12 up to 28 seeds per fruit, for those from trees on “Fepagro C-13” and “Cleopatra” respectively (Table 3). The number of seeds per fruit found in our study is similar or even fewer than those reported in distinct mandarins and hybrids, including “Cravo,” “Nules,” and “Murcott” tangor (Pio et al., 2005; Pacheco et al., 2017). Fruits from the trees on “Fepagro C-13” citrange showed the lowest number of seeds per fruit on most evaluations (Table 3). This is a desirable characteristic, as seedless fruits or fruits with a low number of seeds are preferable to the consumer (Albrigo et al., 2019; Spreen et al., 2020). Although, studies on consumer preference in the United States, suggested that sweetness, shape, acidity, and flavor are more important factors to the purchase decision than the number of seeds (House et al., 2011; Baldwin et al., 2014).

Juice content is another important quality parameter for the commercialization and consumption of citrus fruits. Citrus containing lower juice content than the commercial standards [Organization for Economic Co-operation and Development (OECD), 2010; Companhia de Entrepostos e Armazéns Gerais de São Paulo (CEAGESP), 2011] are depreciated at the fresh and industrial markets as the fruit became tasteless with low levels of organic acids and soluble solids, reducing the saleable weight of the fruit that causes economic loss (Jones and Cree, 1965; Ladaniya, 2008). Fruits from trees grafted on the mandarin rootstocks were among those with the highest juice content in most evaluations, while fruits from trees on “Rangpur” lime exhibited the lowest juice content for this period (Table 3). Regardless of the rootstock, the juice contents of “Emperor” mandarin fruits were above the minimal standard of the international fresh citrus market, which is 33% [Organization for Economic Co-operation and Development (OECD), 2010], in almost all crop seasons. However, fruits solely produced by trees on “Cleopatra” reached this requirement in the 2014 season (Table 3), as this parameter is dependent on several factors that include soil-climate conditions, nutritional balance, field management, and water relations (Figure 1; Castle, 2010; Albrigo et al., 2019). Previous work has confirmed this trend conferred by “Cleopatra” for “Lane Late” sweet orange (Emmanouilidoua and Kyriacoub, 2017). Regarding the Brazilian fresh citrus market, the minimal marketable juice content for mandarin and hybrid fruits is 35% [Companhia de Entrepostos e Armazéns Gerais de São Paulo (CEAGESP), 2011]. Based on this threshold, “Emperor” mandarin trees grafted on most evaluated rootstocks produced fruits that reached this baseline in the 2012, 2013, and 2015 seasons (Table 3). However, in the 2014 and 2016 seasons, almost all “Emperor”-rootstock combinations produced fruits with low juice content, below this standard (Table 3), which evidences the need for better management adoption for “Emperor” mandarin, as fruit thinning and irrigation system that in terms regulate fruit quality.

Although the external appearance of the mandarin fruits is very important, changes in the chemical internal quality of the fruit determine the maturity level (Albrigo et al., 2019). Citrus are classified as non-climacteric fruits and must be harvested when the internal maturity has been achieved, as no further relevant maturation changes will occur in these fruits after harvest (Lado et al., 2014; Albrigo et al., 2019). As mandarin fruit matures, the TSS content increases and the TA levels decrease, in which TSS becomes nearly constant or increases slightly at the late stage of fruit development (Ladaniya, 2008; Albrigo et al., 2019). In general, the balance between sugars and organic acids in juice is the main indicator of mandarin maturity (Ladaniya, 2008; Lado et al., 2014; Goldenberg et al., 2018).

Based on our results, “Emperor” mandarin juice peaked the highest TSS content in the 2012 and 2014 seasons (Table 4). This fact may be related to the climatic conditions, as trees were not irrigated and relied on natural rainfall. Lower rainfall volumes were recorded during these seasons, prior to the harvest time, which may have regulated the fruit quality, particularly in 2012 (Figure 1). According to previous studies, there is an increase in TSS accumulation in fruits of “Satsuma” mandarin trees under water stress, because of the increase in the osmotic potential and sucrose hydrolysis (Yakushiji et al., 1998; Barry et al., 2004). The authors support that this effect is independent of the fruit size and juice content, and is not caused by passive dehydration. However, the water stress can also cause dehydration in the fruit and consequently higher accumulation of TSS (Stenzel et al., 2006), which is supported by the low juice content reported in the 2012 and 2014 seasons (Table 3).

The rootstock also had a large effect on TSS accumulation. Fruits from trees on “Swingle” citrumelo and “Fepagro C-13” citrange showed higher TSS content than those on “Rangpur” lime (Table 4). This may be caused by differences in tree water status influenced by the rootstock (Barry et al., 2004). Previous studies have reported high TSS content in fruits of “Okitsu” satsuma mandarin on “Swingle” citrumelo and “Fepagro C-13” citrange rootstocks (Tazima et al., 2014). Whereas, low TSS scores were found in fruits of “Michal,” “Fallglo,” and “Sunburst” mandarin trees grafted on “Rangpur” lime, supporting our findings in the present study (Mourão Filho et al., 2007; Brugnara et al., 2009). Despite the differences, “Emperor” fruits produced by trees on all tested rootstocks reached TSS above 9%, which surpasses the minimal standard established for the fresh citrus market [Organization for Economic Co-operation and Development (OECD), 2010; Companhia de Entrepostos e Armazéns Gerais de São Paulo (CEAGESP), 2011].

The TA of citrus juices is also an important factor in overall juice quality and in determining the time of harvest (Harding et al., 1940). According to Pereira et al. (2006), the citric acid level in mature mandarin fruits must range between 0.5 and 1.0%. The TA levels recorded for “Emperor” mandarin fruits were close to those obtained for “Clementine” (0.70–1.20%) and “Okitsu” satsuma (0.88–1.03%) mandarins (Georgiou, 2002; Tazima et al., 2014). “Swingle” citrumelo induced the highest TA level to “Emperor” mandarin fruits, while “Rangpur” lime imparted the lowest TA means in most seasons (Table 4). Similar results were reported for “Michal” (Brugnara et al., 2009), “Okitsu” satsuma (Cantuarias-Avilés et al., 2010), and “Oneco” mandarins (Gonzatto et al., 2011). Some studies suggest that the rootstock can influence the fruit maturity stage, by delaying or advancing it, allowing an extension of the commercial season for the canopy cultivar (Stenzel et al., 2006; Morales et al., 2020). The lowest acidity loss exerted by “Swingle” citrumelo associated with the high TSS may prolong the commercial period of the “Emperor” mandarin, by still imparting good TSS/AT at the end of the season, while fruits from the trees on “Rangpur” lime may be tasteless and flat by that time (Morales et al., 2020). Although some consumers do not prefer acidic fruits, the lack of acidity turns the fruit tasteless and flat, unsuitable for fresh consumption (Ladaniya, 2008). A fluctuation in TA level was observed over the evaluated period. “Emperor” mandarin juice showed low acid content in the first three seasons and increased significantly in the last two seasons (2015 and 2016), being higher than 1.12 for all scion-rootstock combinations (Table 4). This was probably related to the HLB infection in the orchard in those seasons (Table 5). The citrus fruits produced by HLB-infected trees usually have disease low TSS and TSS/ratio and high TA (Dagulo et al., 2010; Dala-Paula et al., 2018, 2019).

The acceptability of TSS/TA ratios for the commercialization of mandarin fruits varies according to the target market and usually fluctuates from 7 up to 9:1 (Albrigo et al., 2019). Fruits produced on all scion-rootstock combinations showed TSS/TA ratio higher than 8.5 in the first years of evaluation (Table 4), which meets the standard requirements of the Brazilian fresh citrus market [Companhia de Entrepostos e Armazéns Gerais de São Paulo (CEAGESP), 2011]. On the other hand, only “Emperor” mandarin fruits from trees on “Rangpur” lime reached the minimal standard in the 2016 season (Table 4). The lowest TSS content and the highest TA recorded in the last 2 years of evaluation have contributed to the decrease of the index (Table 4). Although the effect on the content of sugars and acids depends on the rootstock/scion interaction, some rootstocks have the same effect on different cultivars (Albrigo et al., 2019). The high ratio observed for “Emperor” mandarin fruits from trees on “Cleopatra” in this study (Table 4) is consistent with those reported for “Marisol” mandarin (Bassal, 2009) and “Valencia” sweet orange (Bowman et al., 2016).

The TI is an important qualitative parameter for the processing industry, in which higher TI values mean fewer boxes of fruits needed to produce one ton of frozen concentrate orange juice (FCOJ) at 65°Brix, as this index indicates the amount of TSS in a standard citrus box of 40.8 kg (Di Giorgi et al., 1990). Although mandarins are primarily commercialized in the fresh fruit market, due to their deep color and quality, the citrus industry may use mandarin juice to blend with orange or other fruit juices to improve their color and odor/aroma or to sell the juice as single strength (Pérez-López et al., 2006; Albrigo et al., 2019). In our study, the TIs were low in the 2014 and 2016 seasons (Table 4). It may be related to the low juice content reported in these seasons since TI is based on TSS and juice content (Di Giorgi et al., 1990). The technological indices observed for “Emperor” mandarin over the evaluated period were slightly lower than the ones reported by Tazima et al. (2014) for “Okitsu” satsuma mandarin grafted in the same rootstocks.

The natural occurrence of Huanglongbing (HLB) in our experimental orchard has shown that all tested rootstocks combined with “Emperor” mandarin are susceptible to the disease (Table 5). Although there was no statistical difference, “Emperor” trees grafted on “Cleopatra” and “Fepagro C-13” rootstocks had a higher incidence of HLB compared with all other combinations (Table 5). These results corroborate previous reports, in which trees on “Cleopatra” mandarin were the most affected by HLB (Lopes and Frare, 2008; Albrecht and Bowman, 2012). The effect of the disease on fruit quality was evidenced in this study in the last two evaluated years when the infection rate in the grove was higher (Tables 3–5). In general, “Emperor” mandarin juice scored lower for TSS and TSS/TA ratio, but higher for TA (Table 4). The external qualitative parameters, including fruit size and weight, decreased significantly in 2016 compared to the previous seasons (Table 3) which have compromised the marketable value of the fruits. These results are important for the citrus industry as there still have a lack of studies regarding the HLB effect on mandarin fruit quality; however, our findings are in agreement with those reported for sweet oranges, in which the effects of the disease were plenty studied (Dagulo et al., 2010; Liao and Burns, 2012; Massenti et al., 2016; Baldwin et al., 2018; Dala-Paula et al., 2018).

In general, the trees grafted on “Rangpur” lime had the lowest vegetative growth, high yield efficiency, and started fruiting early. However, this scion-rootstock combination produced fruits with lower fruit quality compared with the other tested scion-rootstock combinations. These fruits exhibited low fruit weight, juice content, and TSS. Trees on “Swingle” citrumelo and “Fepagro C-13” citrange showed the lowest scion-rootstock affinity, however, no clear signs of incompatibility were observed in the trees. These rootstocks also induced higher TSS to “Emperor” mandarin fruits. Fruits from trees on “Fepagro C-13” citrange also showed few number of seeds; however, this rootstock induced the lowest yield efficiency. Trees grafted on “Swingle” citrumelo started to bear fruits early and showed high yields over the nine cropping seasons with high yield efficiency. “Cleopatra” and “Sunki” mandarins induced higher juice content for “Emperor” mandarin across the evaluated period. Fruits produced by trees on “Cleopatra” exhibited a higher TSS/TA ratio.

## Conclusion

Rootstocks significantly influenced the tree vegetative growth, fruit yield, and quality of “Emperor” mandarins. Based on our findings, “Cleopatra” and “Sunki” mandarins, “Swingle” citrumelo, and “Fepagro C-13” citrange are more suitable rootstocks for “Emperor” mandarins under the Brazilian subtropical conditions than “Rangpur” lime. Despite inducing low tree size, early fruiting, and high yield efficiency, “Rangpur” lime induced lower fruit quality compared with the other rootstock options. The rootstock choice should be made depending on their specific characteristics and the prevalent interest of the region/market. “Swingle” citrumelo induces early fruiting, high fruit yield and yield efficiency, and good fruit quality with high TSS content. “Fepagro C-13” imparts good fruit quality with a low number of seeds per fruit and high TSS, but low yield efficiency to “Emperor” mandarin. While “Cleopatra” and “Sunki” mandarins induce high juice content. “Cleopatra” also imparts a high TSS/TA ratio to “Emperor” mandarin fruits.

## Data Availability Statement

The original contributions presented in the study are included in the article/supplementary material, further inquiries can be directed to the corresponding author/s.

## Author Contributions

MC: data collection, formal data analysis, and writing—original draft. CN: supervision, writing—review and editing, and resources. DC: formal data analysis and writing—review and editing. RC: formal data analysis and investigation. JB: writing—review and editing. IY: investigation. RL: conceptualization, writing—review and editing, and resources. ZT: conceptualization, supervision, writing—review and editing, funding acquisition, resources, and investigation. All authors approved the submission.

## Conflict of Interest

The authors declare that the research was conducted in the absence of any commercial or financial relationships that could be construed as a potential conflict of interest.

## Publisher’s Note

All claims expressed in this article are solely those of the authors and do not necessarily represent those of their affiliated organizations, or those of the publisher, the editors and the reviewers. Any product that may be evaluated in this article, or claim that may be made by its manufacturer, is not guaranteed or endorsed by the publisher.
